# The Use of Art Observation Interventions to Improve Medical Students’ Diagnostic Skills: A Scoping Review

**DOI:** 10.5334/pme.20

**Published:** 2023-05-23

**Authors:** Anjali Mehta, Steven Agius

**Affiliations:** 1 Education Centre, School of Medicine, The University of Nottingham, Nottingham, United Kingdom

## Abstract

**Introduction:**

Clinical observation skills are fundamental to the practice of medicine. Yet, the skill of looking carefully is rarely taught within the medical curriculum. This may be a contributory factor in diagnostic errors in healthcare. A growing number of medical schools, especially in the United States, have turned to the humanities to offer visual arts-based interventions to foster medical students’ visual literacy. This research aims to map the literature on the relationship between art observation training and diagnostic skills of medical students, highlighting effective teaching methodologies.

**Methods:**

Based on the Arksey and O’Malley framework, a comprehensive scoping review was conducted. Publications were identified by searching nine databases and hand searching the published and grey literature. Two reviewers independently screened each publication using the pre-designed eligibility criteria.

**Results:**

Fifteen publications were included. Significant heterogeneity exists between the study designs and the methods employed to evaluate skill improvement. Nearly all studies (14/15) reported an increase in the number of observations made post-intervention, but none evaluated long-term retention rates. There was an overwhelmingly positive response to the programme, but only one study explored the clinical relevance of the observations made.

**Discussion:**

The review establishes improved observational acumen following the intervention, however, uncovers very limited evidence towards improved diagnostic abilities. There is a need for greater rigour and consistency within the experimental designs, through using control groups, randomisation, and a standardised evaluation rubric. Further research on the optimal intervention duration and the application of skills gained to clinical practice, should be performed.

## Introduction

Medical schools have increasingly engaged with the humanities to train students in the practice of observation skills [1]. Art observation interventions form a subtype of the medical humanities and help establish parallels between fine art concepts and clinical scenarios. Teaching medical students how to observe attentively through describing works of art may help hone their primary observation skills. This in turn can improve their visual literacy and foster diagnostic skills that may not be developed in the standard curriculum [2]. The art of looking carefully to gather evidence, alongside knowledge and experience, is key to forming diagnoses accurately and efficiently [3]. The association between improved observational skills and improved diagnostic abilities needs to be explored further, however, as it cannot simply be assumed.

The ability to examine a patient thoroughly and accurately is fundamental in medicine as it largely contributes to forming a clinical diagnosis. Developing diagnostic confidence and competence can help reduce diagnostic errors, which are estimated to be as high as 10–15% [4] representing the leading cause of preventable harm. Having said that, it is important to caveat that diagnostic error is often not an individual problem but a much more complex systems level issue.

Formal art observation training involves structured and unbiased artwork observation within a museum or art gallery where the sessions are generally led by a clinician and/or an art educator. This intervention requires the student to act as an observer and does not involve direct participation in the creation of an art piece. Currently nearly 70 medical schools offer art-based interventions to their students, mainly in the United States, but also in Canada and Italy [5]. The majority of these are elective modules, with only four mandatory training programmes [1].

Researchers have identified several benefits associated with art observation training, including improved detail perception, as well as greater affinity to pattern and emotion recognition [6]. Additionally, researchers have explored how observation occurs on different levels, relating it to the dual process theory, and how it must be distinguished from inference [7]. Many educators have critiqued the concept of judging the effectiveness of the arts by technical measures, however, arguing that they fail to appropriately determine the impact on the development of both personal and professional qualities of students [8].

We acknowledge that a small number of syntheses evaluating the effectiveness of art observation training in medical education already exist. However, the specific research question and methodology employed in earlier studies significantly differ from this review. The study by Mukunda et al. assesses a wider range of domains, including empathy, teamwork, wellness, and cultural sensitivity [1]. Although it does also address clinical observation, the data extraction for this domain is limited, in comparison to the extraction tool devised for this review. A systematic review by Elbert and ten Cate is over a decade old, and only includes seven studies identified from three electronic databases [6].

With formal art observation training on the rise, it is important for medical educators to understand both effective educational techniques and what effects have been shown in the literature thus far. This will assist in the design of a robust, evidence-based curricula for students and in the implementation of the most effective teaching approaches. This justifies the need to carry out a scoping review, the first in the literature to map the evidence of art observation and diagnostic skills in medical students.

## Methods

This study employed a scoping review methodology, an approach to evidence synthesis which can be used as a rigorous, transparent, and rapid method for systematically mapping out key concepts which underpin a specified research area [9]. It can help provide valuable insights on areas with complexity or which have not been previously reviewed in depth [10], to give an overview of the ‘volume, nature and characteristics of research’ in the field of interest [9]. We utilised the framework outlined by Arksey and O’Malley [9], as it provides a robust foundation for scoping review methodology. We drafted a protocol following the PRISMA Extension for Scoping Reviews (PRISMA-ScR) [11] and adapted using the Joanna Briggs Institute (JBI) guidelines to better suit the purposes of a scoping review [12]. The development of the JBI approach to the conduct of scoping reviews is underpinned by Arksey and O’Malley’s framework, and enhanced through the work of Levac and colleagues [13]. The protocol is outlined in Appendix 1.

### Identifying the research question

This scoping review is designed to map literature describing the impact of formal art observation intervention training on medical students’ diagnostic abilities. A scoping review does not aim to synthesise findings from different studies or formally evaluate the quality of evidence to weight the effectiveness of interventions [14]. We therefore posed the research question: What is the evidence base for the use of art observation interventions to improve medical students’ diagnostic skills?

### Identifying relevant studies

The search strategy followed the three-step process recommended in the 2017 Guidance for the Conduct of JBI Scoping Reviews [12]. Published articles were identified through a comprehensive search of *PubMed*, *EMBASE*, *Scopus*, *Web of Science*, *PsycINFO*, *ASSIA*, *CINAHL Plus*, *Cochrane Library, and ProQuest* without any limits. The full list of databases included within ProQuest can be found in Appendix 2. The most recent search was undertaken on December 24, 2020. Multiple search terms were included to ensure a comprehensive search (See Appendix 3 for search strategies). Grey literature was also searched on both *Open Grey* and *Open DOAR*.

### Study selection

All the articles retrieved from searches were loaded into EndNote and duplicates removed. Two reviewers (AM and SA) independently screened titles, abstracts, and full-text articles for eligibility. Studies were included if: (i) participants were medical students, of any age, gender, ethnicity, and training level; (ii) participants were enrolled in a formal art observation training programme of any duration; and (iii) the main objective of the study was to evaluate the effect of the intervention on diagnostic skills, either directly or indirectly. Studies were excluded if: (i) the full-text could not be obtained; (ii) participants were taking part in another humanities-based intervention, such as narrative writing or theatre performance; or (iii) English translation could not be obtained. To confirm selection process rigour, the reference list of each included study was hand searched to identify any other potentially relevant papers.

### Charting the data

To collect standard information from each study, AM developed a common analytical framework with eleven key points (See Appendix 4) based on the JBI methodological guidance for Scoping Reviews [10] and adapted for this study. This form was then calibrated and tested by SA to ensure that it would allow for all relevant information to be extracted.

### Collating, summarising, and reporting results

AM and SA charted the data independently and captured it in Excel. The information extracted was then compared and any inconsistencies were resolved through re-visiting the article in question. A formal critical appraisal of the quality of the evidence presented in individual studies was not conducted as this is not standard practice in scoping reviews. For illustrating the results of a scoping review, the recommendations provided by Peters et al. [10] were considered. This involved presenting the results in both a tabular and descriptive format, whilst linking it back to the aims of the review.

## Results

**Figure 1:**
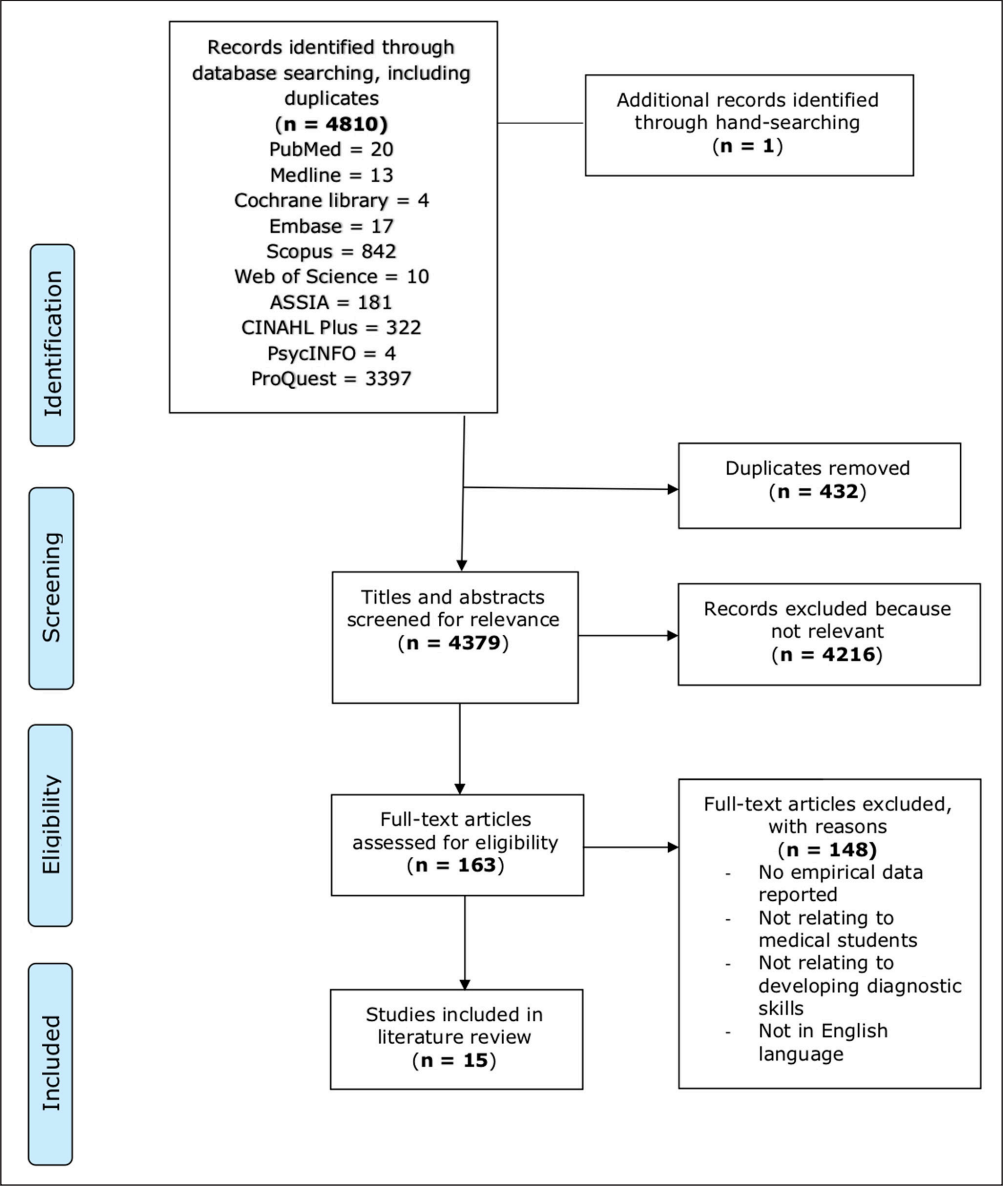
Preferred Reporting Items for Systematic Reviews and Meta-Analyses (PRISMA) flow diagram for the scoping review process [11]. This diagram outlines the steps through which the author selected the studies included in this scoping review.

We identified 4,810 publications of which 15 articles were included (See Figure 1).

### Study population

The supplementary table summarises the key characteristics extracted from all included studies (See Supplementary Table). In ten studies, the intervention was advertised elective [2,15,16,17,18,19,20,21,22,23], and in four studies, the intervention was mandatory [24,25,26,27]. As a result, the sample size varied significantly across the studies ranging from 8 [17]–110 [16] participants. Eight studies included pre-clinical medical students to help them develop the skills needed to observe effectively in upcoming clinical placements [2,17,18,22,25,26,27,28]. Five studies, however, were designed for a different population group – whether that is students in their clinical phase or mixed-year groups [15,19,20,21,23]. The benefit reported in working with participants at a more advanced stage of training included their ability to form a greater number of connections to clinical practice as they were more experienced with patient care [17]. Two studies did not specify their participants’ exact training stage [16,24].

### Experimental designs

Eight studies used a mixed-method approach [2,18,19,22,24,25,27,28]. This combines student satisfaction surveys and numerical data to demonstrate differences in skill following the intervention. For example, Klugman et al. [19] counted the number of words used to describe an artwork, whereas Naghshineh et al. [2] identified thematic categories through evaluating the accuracy and number of clinical observations made.

One study solely took a quantitative approach where data was collected by counting the number of visual findings identified pre- and post-intervention [16]. Finally, six studies employed a qualitative approach [15,17,20,21,23,26], out of which four only relied upon participant self-evaluation to measure the effectiveness of the intervention [17,20,21,26]. Bardes et al. [15] evaluated an improvement in observational skills by comparing the level of precision in image descriptions before and after the intervention. Although this allowed for a qualitative analysis of the narrative, it failed to provide a standard evaluation rubric.

Only six studies [2,16,18,22,25,28] included a control group and four [2,16,19,28] carried out randomisation. None of the studies measured long-term retention rates of the skills as the post-test results were collected shortly after the completion of the programme.

### Art-based education intervention

Intervention designs varied considerably across the studies, ranging from artwork only [17,18,21,22,23,26,27] to a combination of paintings and clinical images [2,15,16,19,20,24,25,28]. Furthermore, an array of artwork styles was used. Shapiro et al. [22] employed a wide range of art genres to help students develop a systematic approach to inspection that can be applied to any given stimuli. Examples include paintings with hidden meanings being used to foster reflection; surreal art to encourage students to look beyond the obvious; and non-representational art to challenge them outside their aesthetic preferences.

Most interventions (n = 12) ran in partnership with a local museum or art gallery, making use of their physical space, while three studies [22,24,27] reported the use of workshops based in a lecture room or clinical environment.

A variety of individuals designed and delivered the interventions (See Table 1). In three studies, delivery was by a medical faculty member with an interest in visual arts [23,24,27]. In the study by Yang et al. [24], most participants agreed that a clinician with sufficient knowledge and experience in art would be a suitable instructor for such an intervention. However, in the study by Jasani et al. [27], some participants reported negative feelings about taking part in a student-led programme, which in turn may have affected engagement levels. On the other hand, three studies relied solely on art educators [17,19,25]. For example, the study by Klugman et al. [19] was led by a museum educator trained in Visual Thinking Strategies, where the results demonstrated an increase in students’ visual observation skills post-intervention. However, they did not examine whether those skills translated into improved clinical practice, an area that could have perhaps been tackled by the involvement of a clinician.

**Table 1:** Individual(s) involved in designing and delivering the intervention.


ART/MUSEUM EDUCATOR ONLY	MEDICAL FACULTY MEMBER ONLY (CLINICIAN/MEDICAL STUDENT)	PARTNERSHIP BETWEEN ART EDUCATOR AND CLINICIAN	NON-SPECIFIED

Agarwal, 2020	Jasani, 2013	Bardes, 2001	Dolev, 2001

He, 2019	Yang, 2011	Karanfilian, 2018	Elder, 2006

Klugman, 2011	Zhao, 2018	Lynch, 2016	Monahan, 2019

		Naghshineh, 2008	

		Schaff, 2011	

		Shapiro, 2006	


In six studies, art educators and physicians collaboratively taught [2,15,18,21,22,28]. For instance, Bardes et al. [15] stated that most students partaking in the intervention, under the guidance of a clinician and an art educator, reported an improvement in their observational, descriptive, and interpretative skills. Shapiro et al. [22] concluded that a joint partnership would be beneficial in terms of promoting interdisciplinary collaboration but would also enable the educators to learn from each other’s skills and strengthen the existing pedagogy. The study by Miller et al. [29] further supported this conclusion, adding that it would reinforce the ‘parallels between medicine’s core clinical skills and art observation’.

The duration of the intervention varied significantly between different studies, ranging from 1 hour [29] up to 20 hours [2] of teaching. There is no agreed number of sessions required to impact on the skillset of students and there are disagreements between various academics on what the ideal duration should be. For instance, Naghshineh et al. [2] concluded that a ‘dose-response’ relationship exists for individuals who engaged in eight or more sessions, compared to those who participated in seven or fewer sessions. However, the study by Klugman et al. [19] demonstrated that even a shortened programme of three sessions only, has the potential to significantly enhance participants’ observation skills.

### Reported outcomes

Most empirical studies [16,18,19,30] simply focused on the number of general observations made pre- and post-intervention. The results indicated an increase in both the time spent observing artwork and percentage of observations made. However, the authors acknowledged that the quality of the observations was not analysed, and it was therefore unknown whether this would translate into gathering more clinically relevant data in patients that may otherwise have been overlooked. One exception is the study by Jasani et al. [27] which reported no significant difference in the mean number of findings identified before and after the intervention.

Only one study measured the number of clinically relevant observations made post-intervention. Agarwal et al. [25] evaluated the impact of VTS workshops on the observation skills of pre-clinical year students. Unlike the other studies, however, they directly focused on the clinical impact of the intervention and ultimately its benefit to medical students. An increase in the number of clinical observations, as well as general patient findings, was reported in the post-intervention group when compared to the control. However, no significant differences in the percentage of diagnostic findings were observed between both groups. These findings question the assumption made as to whether improved observational skills directly translate into ameliorated diagnostic abilities.

## Discussion

We identified a parallel between the process of systematically examining artwork and gathering evidence from a patient’s medical presentation to form a thorough and nuanced understanding. This highlights the importance of the role of observation and considering various interpretations or differentials in order to reach an accurate diagnosis. Although the interventions described have been linked to improving medical student’s observational acumen, the link to improved diagnostic abilities currently has insufficient evidence as only one study investigated this connection.

The review also identifies significant methodological limitations in the published studies, as well as selection bias because of the elective nature of many interventions. This demonstrates the need for greater rigour and transparency in reporting. This suggests that a wider research scope is needed to inform on the efficacy of such interventions, both in relation to clinical practice and on long-term retention through longitudinal studies. This would in turn help justify the cost of resources and time spent running such programmes. A formal mapping exercise has not previously been conducted and allows this review to uncover key concepts in the use of art observation training whilst recommending evidence-based implementation strategies for the future detailed later in this section.

### Population group

It is well established that randomising the allocation of participants to the intervention and control group is essential to increase the probability of equivalence between both sets. Only a fifth of studies included in this review adhered to this principle which means that stratification for gender and other relevant factors, such as background in the arts, was not always considered. As a result, some participants may have carried out previous degrees in the humanities and have greater knowledge and experience in the field, which therefore skews the results collected. To avoid contamination, a possibility would be to only recruit students without any prior exposure to arts-based interventions. Furthermore, over half the studies (60%) introduced art observation training as an elective module, which means that the students who volunteer to take part have an existing interest in the field. Consequently, some of the results may not be generalisable if the programme was mandatory. This is because some students would be taking part simply to meet academic requirements which suggests that they may be less inclined to actively participate.

The inclusion of control groups receiving a non-arts-based intervention is useful to minimise the probability of a Hawthorne effect, and thus increase internal validity [31]. For instance, in the study by Dolev et al. [16], the intervention group visited the Yale Centre for British Art whereas the control group attended clinical tutorial sessions on history taking and physical examination skills. By assigning an activity to both groups, the chances of individuals improving simply because they were subject to an intervention are lowered, which helps to reduce any potential confounding variable [1].

### Programme design

The study by Jasani et al. [27] is the only one that reported no significant difference in observational skills pre- and post-intervention. A reason for this may be due to the flaws in the programme design. For instance, the programme was developed and delivered by a fourth-year medical student with an interest in visual arts which does not substitute for the expertise or knowledge of a professional. Moreover, the intervention was targeted at the whole third-year cohort in a classroom environment within two sessions only. The absence of small group discussions and an expert in the field may have therefore resulted in a lack of student engagement.

Furthermore, the evidence gathered suggests that a short introduction which underlines the educational aims and objectives of the intervention would be beneficial. This is because a small number of students in the study by Jasani et al. [27] demonstrated uncertainty as to how the programme would be relevant to clinical care, with a particular comment stating, ‘I am not quite sure what I learned…Perhaps it will be useful once we get to the floors.’ This suggests that there was insufficient clarity regarding the utility of the sessions provided, which may have affected student level of engagement. It also raises the question as to whether the programme would be of greater benefit once students have entered the clinical phase of their studies. However, the evidence for the intervention to occur later in training is less compelling. This is because the study design becomes more challenging when introduced at a later stage as it is unlikely that the intervention delivered is the sole variable to impact clinical performance [25]. Furthermore, as described in the introduction, observation is a skill that can be taught, and thus requires time and practice to develop. Incorporating the programme early into medical education offers students that opportunity. In addition, medical students, particularly in the early years, often express desire for greater patient contact, which is an element that can be tackled through this intervention [15].

Studies have often combined art observation training with didactics, so the relative contribution of each is difficult to distinguish. For instance, Naghshineh et al. [2] illustrated that there was a direct benefit to integrating clinical teaching within the intervention to improve students’ visual literacy skills. However, a more recent study by Gurwin et al. [32], reported an increase in post-test scores without any complementary clinical component. The authors therefore deduced that art observation training in isolation can foster improved observational skills in medical students. Moreover, the evaluation of single art-based intervention would be useful in determining the relative efficacies of distinct art genres. As a result, this would help inform educators on the style(s) of artwork most appropriate and valuable for the intervention.

### Dose-response relationship

The existing evidence on the ‘dose-response’ relationship of the intervention is inconclusive. Further research on the optimal length of the programme required to enhance clinical observation skills is necessary. This information is important to determine the feasibility of integrating the intervention within the rigid time constraints for medical student learning. In addition, it would help calculate the cost of time for both physicians and art educators, as well as any additional resources required. A greater understanding on this subject would help justify whether the benefits associated with the programme outweigh its limitations.

It is crucial to note that in museums, students had the luxury of time to observe an image and could therefore practise the art of looking slowly and deeply [33]. However, it is not always possible to replicate this approach in a fast-paced clinical environment. The aim of these interventions is to equip students with the set of skills required to observe effectively through challenging one’s assumptions and being aware of one’s own visual blind spots. These fundamental principles can then be further cultivated in clinical practice.

### Evaluation of Art Observation Interventions

In terms of the evaluation method, it is essential to move beyond student self-evaluation surveys to more rigorous assessments on the efficacy of the intervention. Whilst evaluating students’ own perceptions on a potential change in skill set can offer valuable insight into satisfaction and engagement levels, it does not necessarily translate into improved diagnostic skills. Incorporating a pre- and post-test design would enable incremental changes in each student to be measured as baselines assessment measurements have been taken. A combination of both qualitative and quantitative analysis is therefore essential to provide a robust evaluation method.

However, there has been a lack of standardisation and specificity in evaluating skill improvement across the different studies. As a result, Lynch et al. [28] developed a 1-to-5 scoring rubric to specifically measure the change in visual observation skills. Even though this model is promising, it requires further refinement as the examiner feedback indicated that scores of 2–3 and 4–5 were difficult to differentiate between. Karanfilian et al. [18] refined this previous rubric, by increasing clarity as well as describing each rating in relation to others, to measure improvement more accurately. Nevertheless, this scoring system needs to be employed by other educators to further validate its usefulness and efficacy.

### Strengths

A key strength of this scoping review is that it uncovers a total of 8 new published studies in this field which have not previously been included in the existing syntheses. This adds to the existing knowledge base as it offers greater evidence of the breadth of work that has been conducted. It appears to provide the first synthesis of the literature in the field of art observation training to enhance medical students’ diagnostic skills. As well as this, a total of nine different databases were screened to identify relevant data, and no filters were set on publication dates and study designs. This allowed the review to be comprehensive in terms of mapping the range of existing studies and identifying the various methodologies employed.

Furthermore, a second, independent reviewer (SA) screened the full-text articles. The systematic process used to uncover the evidence described has also been documented in a transparent and rigorous manner, to ensure that this review is replicable by fellow researchers. Additionally, this review provides a set of 7 evidence-based recommendations for medical educators and makes suggestions for future scientific research accordingly. Although the use of more rigorous assessment methods has previously been recommended, using a standardised evaluation rubric has not been described in the existing literature syntheses. This uncovers a new conceptual key as this addition may help better illustrate the effectiveness of art-based interventions.

### Limitations

Although the literature search was designed to be as comprehensive as possible, some relevant studies may have been inadvertently missed. Furthermore, studies where the English translation was inaccessible have been excluded which means that the review does not encompass all the international literature available. As a result, the potential influence of studies from non-English speaking countries has been neglected. As per the scoping review guidance, the quality of studies included was not systematically investigated. It is therefore important to consider this lack of discrimination when analysing and extrapolating the results.

### Suggestions for practice

The findings of this scoping review may be used to inform an evidence-based approach to conducting visual art-based interventions for medical students.

- Consider including a control group receiving a non-arts-based intervention and randomise the participants allocated to each group to remove any confounding effects.
- Develop and deliver the programme as a collaborative partnership between both an art educator and an experienced clinician. This might help strengthen the connections between the galleries and the wards, as well as highlight the clinical relevance of such interventions.
- Conduct a multicentre study, with the same intervention to see whether the effects are translatable. This may also maximise the external validity of the study.
- The refined rubric model by Karanfilian et al. [18], may be used to measure the changes in visual observation skills of students. This could ensure standardisation in evaluating skill improvement across the different training programmes.
- Evaluate long-term retention of skills. Future studies should assess the impact of art observation training longitudinally, beginning with first-year students and continuing through to the end of medical school, to measure sustained learning.
- Consider investigating the impact of formal art observation training across the continuum of medical trainees. This would evaluate whether the benefit observed is dependent upon the level of clinical expertise and would help determine the stage at which the intervention is of most benefit.
- Determine whether the skills gained are transferable to clinical practice. As Miller et al. [29] stated, it is not sufficient to leave the learnings in a museum, and future studies should therefore be designed to measure clinical outcomes. A proposed study design would therefore be to divide the training programme into two stages; observation training in an art gallery followed by the application of learnings in a clinical environment.

## Conclusion

This scoping review provides an up-to-date synthesis in the practice of visual art observation training to improve medical students’ diagnostic skills. The included studies offer some evidence that such interventions facilitate the development of observational skills. However, the transfer of these observational skills to improved diagnostic skills has been insufficiently investigated as most studies were limited to the classroom environment and so there is as yet no clear affirmation of a direct link between the two variables. We identified numerous questions waiting to be answered, such as what is the optimal program duration in terms of dose and frequency of training? Which genre(s) of artwork is/are the most effective at teaching key observational skills to medical students? Does an increase in the number of observations made translate into improved diagnostic abilities? We encourage the medical education research community to address these questions through further empirical research.

## Additional Files

The additional files for this article can be found as follows:

10.5334/pme.20.s1 Appendices. Appendices 1 – 4.

10.5334/pme.20.s2 Supplementary Table. A summary of the key characteristics extracted from the studies included in this scoping review.
