# Supplementary material for: The Use of Art Observation Interventions to Improve Medical Students’ Diagnostic Skills: A Scoping Review
Source: Perspect Med Educ. 2023 May 23;12(1):169–78. doi: 10.5334/pme.20 (PMC10215995; doi:10.5334/pme.20)
Supplement: Appendices. — Appendices 1 – 4. [file pme-12-1-20-s1.pdf]

Appendix 1: A Scoping Review Protocol

ADMINISTRATIVE INFORMATION

**Review title**	The Use of Formal Art Observation Interventions to Improve Medical Students' Diagnostic Skills: A Scoping Review
**Amendments**	This study protocol has been written following the PRISMA-ScR guidelines (Tricco et al. 2018), and has been further refined using the Joanna Briggs Institute for conducting scoping reviews (Peters et al. 2015). Any changes following undertaking the review will be explicitly stated and justified in the 'Methods' chapter of the report.
**Funding sources/ sponsors**	No financial support or sponsors are required to complete this review.
**Conflicts of interest**	There are no known conflicts of interest to declare.

INTRODUCTION

**Rationale**	The humanities are becoming increasingly more prevalent in the medical school curricula in order to 'improve the communicative, metacognitive, and observation skills' of students (Rana, Pop, and Burgin 2020). These educational interventions are developed to help participants explore knowledge from a perspective that is different to the traditional scientific approach, hence encouraging a holistic professional formation.<br><br>Clinical observation is a fundamental part of medicine, yet its teaching is rarely included in the medical school curricula. Contrary to the belief that diagnostic skills are solely developed through years of practice, there has been increasing evidence that these skills can in fact be nurtured using the right interventions.<br><br>Arts-based interventions in health care education may, amongst other things, help improve clinical diagnostic skills. At the moment such programs exist in a number of medical schools globally, however these are mostly advertised as elective modules. A preliminary search on this topic indicated significant literature available, however there is no scoping review that exists in this field to date. This further emphasises the importance of this study to map the current evidence in order to adequately inform on future research needed.
**Objectives**	1. To map the existing literature in order to understand the relationship between art observation training and diagnostic skills of medical students.<br>2. To explore how the findings of this review can influence the way the current medical curriculum is designed to benefit both students and their patients.<br>3. To identify the gaps in the current literature and make recommendations about future research accordingly.

**Key words**	A. **Scoping reviews** are becoming an increasingly popular tool to systematically map the breadth of evidence in a particular body of literature. Although no universal scoping study definition exists, Levac et al. (2010) define it as the 'synthesis and analysis of a wide range of research and non-research material to provide greater conceptual clarity about a specific topic.' Unlike a systematic review, the purpose of a scoping study is not to establish the quality of the evidence, but instead to identify gaps in knowledge and trends in the current evidence in order to underpin future research.<br><br>B. While the definition of **art** is widely disputed and has evolved over time, in its broad form, it may be considered as 'a form of communication' and 'an act of expressing feelings, thoughts, and observations' (Brewminate 2017). Art encompasses multiple modalities, including but not limited to literature, poetry, music, dance, and drama. This review focuses on 'visual art' which is appreciated through sight and opens a window into exploring human experience.<br><br>**C.** **Observation** is simply the 'art of seeing' (Shapiro, Rucker, and Beck 2006). It illustrates the ability to gather information from a primary source and derive meaning in what is inspected from an unbiased point of view. This same construct has also been referred to as 'visual literacy' by Naghshineh et al. (2008) and 'pattern recognition' by Shapiro et al. (2006).<br><br>D. **Diagnostic skills** rely on utilising observations in the context of domain specific knowledge and experience. In order to do so, one must 'recognise both the subtle and obvious visual details' that underpin the clinical presentation to form accurate diagnoses (Dolev, Friedlaender and Braverman 2001).<br><br>**E.** A **medical student** is an individual who is following a course of study at a university in order to qualify as a physician. This study will look at medical students from both pre-clinical and clinical years globally.

METHODS

**Inclusion criteria**	**Types of participants**	Participants must be medical students, however they can be at any stage of their education, whether that is the pre-clinical or clinical phase. As well as this, they can be of any age, gender, and ethnicity as this will make the study more comprehensive. The background characteristics of the participants, in terms of medical conditions or socio-economic background for instance, is not relevant for this purpose.
	**Concept**	The participants must be taking part in a formal art observation training programme, although this can be of any specified length of time. The main objective of the study should be evaluating the effect of the training programme on diagnostic skills, either directly

		or indirectly. For instance, a direct relationship can be seen as improvements in observation skills or reflective and analytical thinking. On the other hand, an Indirect relationship can be classified as a development in communication skills, teamwork, listening skills or even empathy. Including these various aspects add breadth to the scoping review and ensures a more comprehensive mapping of the existing literature.
	**Context**	All studies, whether conducted nationally or internationally will be included in the review. As well as this, there will not be a limit to the publication dates. The specific setting of the study, whether that is a lecture theatre or a local museum, will vary depending on the facilities available, but this will not affect the eligibility criteria.
**Exclusion criteria**	Studies will be excluded if they have any of the following characteristics:<br>- Studies where the participants are not medical students<br>- Studies where the participants are taking part in another humanities-based curricula intervention, such as narrative writing or theatre<br>- Data collected is not relevant, either directly or indirectly, to diagnostic skills<br>- Articles where the full text cannot be obtained<br>- Studies where the English translation cannot be obtained	
**Information sources**	In order to map the existing and emerging evidence, any literature relevant to this subject will be screened and no filters will be added. All research designs, as well as websites pages, will be considered.	
**Search strategy**	The search strategy will follow the three-step process recommended in the 2017 Guidance for the Conduct of JBI Scoping Reviews (Peters et al. 2015).<br><br>The first step is an initial limited search of NUsearch and Google Scholar.<br>Following this, the key words that appear in the title and abstract of the studies will be analysed, as well as the index terms used to describe the article.<br><br>A second search will then be carried out using all the identified keywords and index terms across all included databases. This includes:<br>- PubMed<br>- Cochrane Library<br>- EMBASE<br>- Scopus<br>- Web of Science<br>- ASSIA<br>- CINAHL Plus<br>- ProQuest<br>- PsycINFO<br>- Open Grey and Open DOAR (grey literature)	

	In the third step, the reference list of selected studies will be searched to identify any additional literature. Ideally, the bibliography of all identified studies should be searched for, however, due to the time constraints of the project, this will be limited to the studies that are included in the review. As well as this, the citation list of each included study will also be searched to minimise the risk of excluding relevant studies.<br><br>In the aim of conducting a comprehensive search on the topic area, grey literature will also be consulted. This will be done through accessing NUsearch for relevant conference papers, theses, and dissertations. As well as this, to identify any ongoing clinical trials, a search on CENTRAL will be carried out.
**Study selection**	All search result citations will be uploaded into EndNote software for ease of reference and tracking. This software will also enable all the duplicates to be deleted.<br><br>Firstly, the title and abstract of each study will be analysed to identify the ones that are directly relevant. If a study's relevance is not clear from the abstract, the whole paper will be read. As explained by Badger et al. (2000), 'abstracts cannot be assumed to be representative of the full article', hence the full text will be studied for chosen articles.<br><br>The predefined inclusion criteria will be strongly referred to when selecting articles which are to be included in the final review.
**Data collection process**	In a scoping review, data collection is known as 'extraction and charting'. According to Ritchie and Spencer, charting is a technique used to synthesize and interpret qualitative data according to key issues and themes.<br><br>In order to collect standard information from each study, a common analytical framework with eleven key points has been developed. This structure was taken from the Joanna Briggs Institute methodological guidance for Scoping Reviews (Peters et al. 2015) and has been adapted to fit the purpose of the study. Any further refinement during the data extraction process will be noted and justified in the methodology.<br><br>1. Author and year of publication<br>2. School (country)<br>3. Museum partnership<br>4. Academic staff involved<br>5. Study population and sample size<br>6. Number of sessions<br>7. Total number of hours<br>8. Methods/ intervention type<br>9. How outcomes are measured<br>10. Control group?<br>11. Key findings<br><br>All the data collected will then be entered into the database programme Excel.
**Presentation**	The process through which studies will be included and excluded in

**of the results**	the review will be presented in the form of a flowchart. Furthermore, as the objective of this scoping review is to understand the impact of an art observation training programme on the diagnostic abilities of medical students, it may be useful to map the data collected in a tabular form. As well as this, a narrative summary will accompany the results to illustrate how this relates back to the objectives of the review.<br>Having said this, the exact framework will be developed further along the process when there is greater clarity on the contents of the included studies.
**Risk of bias (quality) assessment**	As per the proposed framework of Arksey and O'Malley (2005), as well as the Joanna Briggs Institute methodological guidance for Scoping Reviews (Peters et al. 2015), a quality appraisal for the selected studies will not be carried out.
**Limitations**	Only articles where an English translation is available will be used, so that means that there is a possibility to miss out of valuable literature.<br>Moreover, the quality of studies will not be considered, which therefore affects the reliability of the conclusions drawn.

NOTE: For citations in the protocol, please refer to the bibliography.

Appendix 2: Full list of databases included within ProQuest at the University of Nottingham

1. ProQuest dissertations & theses A & I.	2. ProQuest historical newspapers. Chicago

	defender.
3. ProQuest historical newspapers. New York times.	4. ProQuest historical newspapers. Pittsburgh courier.
5. Education collection.	6. Patrologia Latina database.
7. Linguistics collection.	8. Kafkas Werke: kritische Kafka-Ausgabe des S. Fischer Verlages bei ProQuest.
9. International newsstream.	10. ProQuest historical newspapers. Los Angeles times.
11. ProQuest Historical Newspapers. Washington post.	12. U.S. newsstream.
13. Sociology database.	14. The digital library of classic Protestant texts.
15. ProQuest natural science collection.	16. ProQuest historical newspapers. The Guardian and The Observer.
17. Agricultural & environmental science collection.	18. Education in video.
19. Advanced technologies & aerospace collection	20. International bibliography of the social sciences: IBSS.
21. Technology collection.	22. Biological science collection.
23. Politics collection.	24. SciTech premium collection.
25. Early European books.	26. Social science premium collection.
27. Science database.	28. Environmental science index.
29. Criminology collection.	30. U.K. parliamentary papers.
31. Linguistics database.	32. Worldwide political science abstracts.
33. Sociology collection.	34. Materials science & engineering collection.
35. Library & information science collection.	36. The Gerritsen collection of Aletta H. Jacobs.
37. Library science database.	38. The Vogue archive.
39. Social science database.	40. The Kissinger transcripts: a verbatim record of U.S. diplomacy, 1969-1977 / Henry Kissinger; project director William Burr
41. Education database.	42. Sociological abstracts.
43. Political science database.	44. Earth, atmospheric & aquatic science database.
45. Engineering index.	46. National criminal justice reference service (NCJRS) abstracts database.
47. Criminal justice database.	48. Applied social sciences index and abstracts: ASSIA.
49. British periodicals.	50. Literature online.
51. Policy file index.	52. Library and information science abstracts: LISA.
53. ABI/INFORM global.	54. PAIS index.
55. ProQuest historical newspapers. The Times of India.	56. Linguistics and language behavior abstracts: (LLBA).
57. Periodicals archive online.	58. EconLit.
59. Acta sanctorum: the full text database.	60. Journal archives
61. Black freedom struggle in the United States: a selection of primary sources.	62. EEBO: early English books online.

Appendix 3: Search strategy applied to respective databases

This is a table of the search strategies used on the various databases. The fundamental structure is taken from the search strategy chosen above, but it has been modified slightly depending on the databases used.

DATABASE	SEARCH STRATEGY	RESULTS GENERATED	ADDITIONAL COMMENTS
PubMed	(art OR artwork* OR "visual art*" OR "fine art" OR "art course" OR "art in medicine") AND ("medical student*" OR "medical undergraduate" OR "student doctor") AND (observation OR "art observation" OR "observation skill*" OR "observation training" OR "visual thinking strategies" OR "visual literacy" OR "pattern recognition") AND ("diagnostic skill*" OR diagnosis* OR "clinical diagnostic skill*" OR "visual diagnostic skill*")	33	-
Cochrane library	(art OR artwork* OR painting OR "visual art*" OR "fine art" OR "art course" OR "art in medicine" OR museums NOT fertility) AND ("medical student*" OR "medical undergraduate" OR "student doctor" OR medical) AND (observation OR "art observation" OR "observation skills" OR "observation training" OR "visual thinking strategies" OR "visual literacy" OR "pattern recognition") AND ("diagnostic skills" OR diagnosis* OR diagnostic OR "clinical diagnostic skill*" OR "visual diagnostic skill*" OR "clinical competence") AND ("medical humanities" OR "humanities in medicine" OR "medical education" OR "medical curriculum" OR "narrative medicine")	4	-
EMBASE	(art OR artwork* OR "visual art*" OR "fine art" OR "art course" OR "art in medicine") AND ("medical student*" OR "medical undergraduate" OR "student doctor") AND (observation OR "art observation" OR "observation skill*" OR "observation training" OR "visual thinking strategies" OR "visual literacy" OR "pattern recognition") AND ("diagnostic skill*" OR diagnosis* OR "clinical diagnostic skill*" OR "visual diagnostic skill*")	17	-
Scopus	(art OR artwork* OR "visual art*" OR "fine art" OR "art course" OR "art in medicine") AND ("medical student*" OR "medical	842	-

	undergraduate" OR "student doctor") AND (observation OR "art observation" OR "observation skill*" OR "observation training" OR "visual thinking strategies" OR "visual literacy" OR "pattern recognition") AND ("diagnostic skill*" OR diagnosis* OR "clinical diagnostic skill*" OR "visual diagnostic skill*")		
Web of Science	(art OR artwork* OR "visual art*" OR "fine art" OR "art course" OR "art in medicine") AND ("medical student*" OR "medical undergraduate" OR "student doctor") AND (observation OR "art observation" OR "observation skill*" OR "observation training" OR "visual thinking strategies" OR "visual literacy" OR "pattern recognition") AND ("diagnostic skill*" OR diagnosis* OR "clinical diagnostic skill*" OR "visual diagnostic skill*")	10	-
ASSIA	(art OR artwork* OR "visual art" OR "fine art" OR "art course" OR "art in medicine") AND ("medical student*" OR "medical undergraduate" OR "student doctor") AND (observation OR "art observation" OR "observation skill*" OR "observation training" OR "visual thinking strategies" OR "visual literacy" OR "pattern recognition") AND ("diagnostic skill*" OR diagnosis* OR "clinical diagnostic skill*" OR "visual diagnostic skill*")	181	-
CINAHL plus	(art OR artwork* OR "visual art" OR "fine art" OR "art course" OR "art in medicine") AND ("medical student*" OR "medical undergraduate" OR "student doctor") AND (observation OR "art observation" OR "observation skill*" OR "observation training" OR "visual thinking strategies" OR "visual literacy" OR "pattern recognition") AND ("diagnostic skill*" OR diagnosis* OR "clinical diagnostic skill*" OR "visual diagnostic skill*")	322	-
PsycINFO	(art OR artwork* OR "visual art" OR "fine art" OR "art course" OR "art in medicine") AND ("medical student*" OR "medical undergraduate" OR "student doctor") AND (observation OR "art observation" OR "observation skill*" OR "observation training" OR "visual thinking strategies" OR "visual	4	-

	literacy" OR "pattern recognition") AND ("diagnostic skill*" OR diagnosis* OR "clinical diagnostic skill*" OR "visual diagnostic skill*")		
ProQuest	(art OR artwork* OR "visual art" OR "fine art" OR "art course" OR "art in medicine") AND ("medical student*" OR "medical undergraduate" OR "student doctor") AND (observation OR "art observation" OR "observation skill*" OR "observation training" OR "visual thinking strategies" OR "visual literacy" OR "pattern recognition") AND ("diagnostic skill*" OR diagnosis* OR "clinical diagnostic skill*" OR "visual diagnostic skill*")	3397	Filter set: limited to full text articles only

Appendix 4: Data extraction form

The table below illustrates the charting form used to extract the relevant data from each study.

1. First author + year of publication
2. School (country)
3. Museum partnership
4. Academic staff involved
5. Study population and sample size
6. Number of sessions
7. Total number of hours
8. Methods/ intervention type
9. How outcomes are measured
10. Control group?
11. Key findings that relate to the review question
