# Supplementary material for: The Use of Art Observation Interventions to Improve Medical Students’ Diagnostic Skills: A Scoping Review
Source: Perspect Med Educ. 2023 May 23;12(1):169–78. doi: 10.5334/pme.20 (PMC10215995; doi:10.5334/pme.20)
Supplement: Supplementary Table. — A summary of the key characteristics extracted from the studies included in this scoping review. [file pme-12-1-20-s2.pdf]

Supplementary Table. *A summary of the key characteristics extracted from the studies included in this scoping review. Where possible, descriptions of methodology by author(s) have been retained within the table to ensure accuracy of representation. Quotation marks are used to denote this practice.*

**First author, year**	**School (country)**	**Museum partnership**	**Academic staff involved**	**Study population**	**Number of sessions**	**Total number of hours**	**Methods/ intervention type**	**How is the outcome measured?**	**Control group?**
**Agarwal, 2020**	Department of Medicine, University of Miami Miller School of Medicine (United States)	Lowe Art Museum at the University of Miami	Museum educator	-Intervention group: 41 first-year students in the MD/MPH program<br>-Comparative group: 60 first-year MD program medical<br>-Total: n = 101	2	6	- 'Baseline survey assessing for socio-demographic characteristics and prior clinical or humanities training'<br>- 'Timed written response portion to clinical images' both before and after intervention<br>-VTS workshop	-Pre-test and post-test<br>- Quantitative data: 'Analysis of Variance used to analyse the differences between the comparative and intervention group means for each theme, at both baseline and post-intervention.'<br>- Qualitative data: 'A general inductive approach used to analyse and identify themes'	Yes

Key findings

- 'VTS increased the mean number of words provided by students to describe clinical images and the time spent' analysing them.
- 'An increase in the number of general observations and the number of clinically relevant observations' was also increased when compared to a comparative group.

<table>
<tr><td>Bardes, 2001</td><td>Weill Cornell Medical College (United States)</td><td>The Frick Collection, a private art museum in New York</td><td>Medical faculty member and art educators</td><td>8 medical students from first, second-, and fourth-year classes</td><td>3</td><td>Information not provided</td><td>Observation of painted portraits followed by examination of patients' faces photographs.</td><td>-Pre-test and post-test measurement
-Questionnaire designed to assess their appraisal of the experience.</td><td>No</td></tr>
<tr><td colspan="10">Key findings
'This programme, well-received by students and faculty, appeared to help the students not only in improving their empirical skills in observation, but also in developing increased awareness of emotional and character expression in the human face.'</td></tr>
<tr><td>Dolev, 2001</td><td>Yale University School of Medicine (United States)</td><td>Yale Centre for British Art (YCBA)</td><td>Information not provided</td><td>-'In 1998-1999: 90 medical students randomized to a control group (n=30), an intervention group (YCBA, n=30), or a lecture group (n=30).'

- 'In 1999-2000: 86 students randomly assigned to the control group (n=35) or the YCBA group (n=51).'</td><td>Information not provided</td><td>Information not provided</td><td>-Control group: clinical tutorial sessions on 'history taking and physical examination skills.'
-'Lecture group: anatomy lecture on abdominal radiographic images.'
-Intervention group: YCBA program, where 'each student studies a preselected painting for 10 minutes before describing it in detail to their group'. Solely based on visual evidence.</td><td>-A pre-test–post-test study design.
- Statistical analysis performed using the SPSS program and Microsoft Excel with analysis of variance or 2-tailed t-tests
→ quantitative data collected</td><td>Yes</td></tr>
<tr><td colspan="10">Key findings
- Pre-test scores did not significantly differ in both years
- Post-test scores differed significantly between groups in both years; 'YCBA group had significantly higher mean post-test percentage improvement scores.'</td></tr>
</table>

<table>
<tr><td>Elder, 2006</td><td>Department of Family Medicine, University of Cincinnati, Cincinnati, OH, (United States)</td><td>Cincinnati Art Museum</td><td>Information not provided</td><td>17/19 second-year medical students</td><td>Information not provided</td><td>Information not provided</td><td>Elective course titled 'Art of Observation' (AOO)
No specific methodology described.</td><td>Qualitative survey-based evaluation. Online evaluation consisting of eight questions to form themes.</td><td>No</td></tr>
<tr><td colspan="10">Key findings
3 themes identified:
1. 'The AOO positively influenced clinical skills.'
2. 'Both art museum exercises and a clinical preceptorship were necessary to achieve those skills.'
3. 'The AOO led to a sense of personal development as a physician.'</td></tr>
<tr><td>He, 2019</td><td>Optional elective at the University of Texas Southwestern Medical Centre (United States)</td><td>Dallas Museum of Art (DMA).</td><td>Art educator</td><td>65 medical students in 1st, 2nd and 3rd year</td><td>7</td><td>14</td><td>Guided museum visits. Students must attend at least 5 sessions to receive transcript credit.
No additional detail provided.</td><td>Qualitative approach:
- 'Evaluations contained open-ended questions that asked students to reflect upon their experiences.'
- 'Used grounded theory to generate a thematic codebook, then employed axial coding to discover thematic relationships.'</td><td>No</td></tr>
<tr><td colspan="10">Key findings
3 main findings:
1. 'Enhanced observation skills: students developed the ability to synthesize a compelling narrative in addition to learning technical skills.'
2. 'Improved physician socialization: students reported enhanced self-awareness, increased tolerance of ambiguity, and development of a humanistic view of medicine.'</td></tr>
</table>

3. ‘Reduction in burnout symptoms: students reported an enhanced sense of well-being after each session.’									
**Jasani, 2013**	Rush University Medical Centre, (United States)	Classroom	Exercise was designed and led by a fourth-year medical student with interest in the visual arts	110 3rd year medical students (just prior to the start of clerkship)	2	3	-Guided through 8 images and discussion on 8 fine art images.<br>-Used both representational and non-representational art.	-Pre- and post-test and evaluation survey	No
**Key findings**<br>- ‘Mean number of observations between pre- and post-tests was not significantly different.’<br>- Qualitative analysis showed that ‘students felt they increased their mindfulness and skills regarding clinical observations.’<br>- ‘Exercise can be replicated without specially trained personnel or art museum partnerships.’									
**Karanfilian, 2018**	Rutgers Robert Wood Johnson Medical School (United States)	Rutgers University Zimmerli Art Museum.	Art educator and physician	72 First year medical students	3	3	Experimental group: 3 art observation sessions/ 3 clinical observation lectures and 2 podcasts.	-Pre-test and post-test<br>-Survey	Yes
**Key findings**<br>- ‘The modified scoring rubric enabled more accurate and consistent scoring of narrative responses pre- and post- intervention.’<br>- ‘Participation in this training program led to more detailed descriptions of clinical images, as measured by the scoring rubric.’ → Statistically significant increase in the experimental group’s scores between the pre-test and post-test compared to the control group.<br>- Positive student feedback received on the training programme.									
**Klugman, 2011**	University of Texas Health Science Centre (United States)	McNay Art Museum	Museum educators who are trained in VTS	32 students in total:<br>-18 medical students from first-, second- and third-year<br>-14 nursing students (both undergraduate and graduate)	3	4.5	Randomised into groups. Each group visited the same 3 works of art per week in rotation.<br>-Week 1 objective: look at and describe without interpreting meaning.<br>-Week 2 objective:	-Pre and post-test evaluations on SurveyMonkey.<br>-Quantitative data: ‘Students took two standardized tests, Geller and colleagues' variation of	No

							describe and interpret what you see.<br>-Week 3: look at less representational works to describe what they see, focusing on interpreting the emotional quality in works	Budner's Tolerance of Ambiguity Scale and the Communication Skills Attitudes Scale.'<br>- Qualitative data: Free responses to art and patient images.	
**Key findings**<br>- 'Students significantly increased the amount of time they spent looking at art and patient images, the number of words they used to describe art and patient images, and the number of observations made.'<br>- 'Females increased the time spent observing significantly more than did males.'<br>- 'Students significantly increased their tolerance for ambiguity and positive views toward health care professional communication skills.' → Shows that a short course of three sessions can significantly increase students' visual observation skills.									
**Lynch, 2016**	Rutgers Robert Wood Johnson Medical School (United States)	Rutgers University Zimmerli Art Museum	Researcher, the art educator, and the clinician	33 first- and second-year medical students<br>→ randomised	6	?	- Group A: 'Attended 3 art educator-led sessions at the museum in the fall. Attended 3 physician-led clinical sessions in the spring.'<br>- Group B: 'Attended 3 physician-led clinical sessions in the fall. Attended 3 art educator-led sessions at the museum in the spring.'<br>- Group C: No intervention given (control)	-Pre- and post-intervention test on descriptions of clinical images<br>- 'Scored by two reviewers using a 1-to-5 scoring rubric developed for this study.'	Yes

**Key findings**<br><br>- 'Results indicated that participants improved visual observation skills, and a satisfaction survey indicated they enjoyed participating.'<br>- 'No significant differences were found between group A (museum experience first) and group B (clinician experience first), so cannot state whether training order matters.'									
**Monahan, 2019**	University of Illinois at Chicago (United States)	Interprofessional education Art Applications Workshop	Non-specified	'104 medicine, nursing and psychology students. Assigned to teams of six, with a mixture of students from each discipline.'	3	3	-Session 1: Students attend an art lecture and workshop; analyse 'paintings and learn visual assessment techniques.'<br>-Session 2: 'Student teams observe paintings in a museum setting and write observations in case notes.'<br>-Session 3: 'Student teams apply visual assessment techniques to standardized patient interviews, write patient histories, assessment notes.'	-Post-survey self-evaluations	No
**Key findings**<br>- '91% of the students agreed/strongly agreed that they are better at visual observation.'<br>- '92% of the students agreed/strongly agreed that they improved their communication skills in listening and encouraging the ideas and opinions of other team members.'<br>- '91% of the students agreed/strongly agreed that they are more confident in communicating with students from different disciplines.'<br>- '97% of the students agreed/strongly agreed that they are more confident in collaborating with students from different disciplines as a result of the workshop.'									
**Naghshineh, 2008**	Harvard Medical and Dental School (United States)	Boston Museum of Fine Arts (MFA)	Art educators trained in Visual Thinking Strategies	-24 pre-clinical student in intervention group	8	20	- 'Students practiced inspecting, verbally describing, interpreting, and	Pre-post-test: 'All students sat a 1-hour written visual	Yes

			and physicians	- 34 students in control group → randomised			actively building on others' analyses of artworks.'<br>- Opportunity to take part in optional live art session from a model with professional art instruction.<br>- 'Students also had to complete weekly assignments, including focused readings and brief visual training exercises.'	skills examination assessing the accuracy and number of clinical image observations'. Mean change in score calculated for each image.	
**Key findings**<br>- 'The mean frequency of observations on the pre-course visual skills examination did not differ among intervention and control students.'<br>- The 'intervention group had a far greater pre- to post-test change in observational accuracy than the controls.'<br>- A 'dose-response' relationship between number of sessions attended and improvement was found for those who attended eight or more sessions, compared to participants who attended seven or fewer sessions.									
**Schaff, 2011**	The Keck School of Medicine (KSOM) of the University of Southern California (United States)	Los Angeles' Museum of Contemporary Art (MOCA)	'Two clinician educators who have expertise in the visual arts and literary studies, and MOCA's director of education.'	30% of Year 2 students taking part in the 'Introduction to Clinical Medicine (ICM) program completed the Art and Medicine Focus Experience.'	1	3	-Warm up exercise: Students observe a group of non-objective or nonrepresentational works of art.<br>-2nd step: 'Encourage students to reflect on and understand the progress of their thinking and observation processes.'<br>-3rd step: 'Initiation of guided conversations	Evaluation post-intervention	No

							about particular art works.' -4th step: 'Groups of students explore a work of art on their own, noting and then publicly sharing their group process, the questions that developed, and how the group answered them.'		

Key findings

- 'The open-ended meanings embodied by contemporary art allow students to develop a resistance to closure and an ability to manipulate and play with ideas.'
- 'Observing and interpreting contemporary art through a constructivist lens allows medical students to try on various interpretations simultaneously and to discard or transform those ideas as they collaboratively create a web of possibilities about interpretation and significance.'
- 'The students rated the experience's fulfilment of its objectives very highly' → Experience overwhelmingly positive.

**Shapiro, 2006**	The School of Medicine, California (United States)	University of California, Irvine, School of the Arts	Arts-based sessions conducted by 'a faculty member from the School of the Arts and the clinically based sessions by a faculty member from the School of Medicine.'	38 students in Year 3. Divided into 3 groups: -Group A (n=15) trained using clinical photographs and cases. -Group B (n = 11) trained using art. - Group C (n=14) trained using a mixed media format of art and dance.	3	6	Used a range of artwork to develop a systematic process of observation: -Less accessible artwork (e.g.: non-representational art) -Paintings with hidden meanings (to encourage reflection) -Paintings that are outside students' aesthetic preferences (e.g.: surreal art).	'Post-session group interview comments from students, written feedback from students, participant observations, and instructor debriefings.' → 'Multiple sources allowed for data triangulation.'	Yes

<table>
<tr><td colspan="10">Key findings
- ‘The clinically based teaching appeared to be an effective way to teach about disease, while the arts-based method was a useful way to teach about illness.’
- ‘The 2 teaching approaches studied were naturally complementary, and justify the growing inclusion of arts-based pedagogical tools and approaches in medical education.’</td></tr>
<tr><td>Yang, 2011</td><td>Changhua Christian Hospital (Taiwan)</td><td>At the hospital, images shown on slides</td><td>Programme developed by an individual who is both a senior physician and an artist</td><td>110 of the medical students. Compulsory module required.</td><td>1</td><td>4</td><td>-Step 1: ‘Discuss a patient-related painting.’
-Step 2: ‘Use of stories, histories and images to increase motivation.’
-Step 3: ‘Learning to interpret paintings.’
-Step 4: ‘Interpret medicine related paintings.’
-Step 5: ‘Interpret paintings relating to human suffering and relate to humanitarianism.’
-Step 6: ‘Discuss topics in other humanities fields.’
-Step 7: ‘Repeat discussions on the same patient-related painting.’
- Step 8: ‘Discuss the values and meaning with a summary made.’</td><td>- Pre- and post-lecture discussion
- Written feedback by students at the end of the programme
- Records written by students on what they observed</td><td>No</td></tr>
<tr><td colspan="10">Key findings
- ‘Improved visual diagnostic skills in medical students who participated in art observation workshops through systematic visual training using representational paintings.’
- ‘Improve the skills of observation, listening and communication and help to build teamwork and enhance empathy and stress reduction.’</td></tr>
</table>

**Zhao, 2018**	McMaster University, Canada	CanadiEM.org, a medical education blog, developed a new series called Spot the Diagnosis!	- 'Series are selected based upon the author's art history knowledge, resources found using an online search, and/or suggestions made by other healthcare professionals.'<br>- 'The accompanying blog post is researched and written by a medical student and peer-reviewed by another medical student and physician.'	Accessible to anyone with an internet connection. Mainly targeted at healthcare learners	N/A	N/A	'Six Spot the Diagnosis! posts have been published' (uploaded monthly). 'Each of which begins with the selection of a piece of fine arts that showcases a potential medical diagnosis and a blog post outlining an interpretation of the work informed by observations, historical reports, and medical evidence. The answers to questions are hidden under drop-down formatting to allow viewers to arrive at their own answers first. Promotion occurs on site, via email, word-of-mouth, and social media. Viewership is tracked using Google Analytics (GA).'	A survey for readers is planned to assess who, how, and why readers use the series.	N/A

Key findings

- 'In the first 30 days of publication, each post in the series was viewed 1582 ± 401 times.'
- Results are not available prior to abstract submission.
